# Bitumen Emulsion—Mineral Surface Interactions: An NMR Study on the Interface Layer Composition

**DOI:** 10.1002/mrc.70056

**Published:** 2025-11-14

**Authors:** Andrei Filippov, Hilde Soenen, Oleg N. Antzutkin

**Affiliations:** ^1^ Chemistry of Interfaces, Department of Civil and Environmental Engineering Luleå University of Technology Luleå Sweden; ^2^ Nynas N.V. Antwerp Belgium

**Keywords:** bitumen emulsion, interface composition, mineral surface, NMR

## Abstract

Compositions containing bitumen emulsions and solids have many applications, such as soil stabilization, cold mix asphalt preparation, dust binding, surface dressing and slurry sealing. Therefore, the interaction of bitumen with minerals is of great interest in science and applications. In the interaction of bitumen emulsion with a mineral surface, one of the processes that influence the properties of the bitumen–mineral composition is the formation of interface layers. Nuclear magnetic resonance can provide insights into the interactions of bitumen and minerals at the molecular level. However, the presence of magnetic constituents in most solids as well as the background magnetic field gradients at the interface does not allow the use of NMR to its full potential. In this work, we describe a ^1^H NMR approach to study the interface layer formed by a specified bitumen emulsion in the presence of non‐magnetic as well as magnetic minerals. This approach is based on the consecutive flushing off of “bulky” components of the bitumen emulsion and the following extraction of the surface layer material, which can then be analyzed by NMR spectroscopy. As a proof of concept, this technique was tested on samples prepared using a bitumen emulsion and four different silicate minerals. The ^1^H NMR study showed that interfacial layers may accumulate asphaltenes adsorbed to mineral surfaces to a different extent depending on the specific elemental composition of minerals. More asphaltenes were detected in the interfacial layers on surfaces of studied minerals with a higher content of calcium.

## Graphical abstract

We describe a ^1^H NMR approach to study the interface layer formed by a specified bitumen emulsion in the presence of non‐magnetic as well as magnetic minerals. This approach is based on the consecutive flushing off “bulky” components of the bitumen emulsion and the following extraction of the surface layer material, which can then be analyzed by NMR spectroscopy. The study showed that interfacial layers may accumulate asphaltenes adsorbed to mineral surfaces to a different extent depending on the specific elemental composition of minerals.

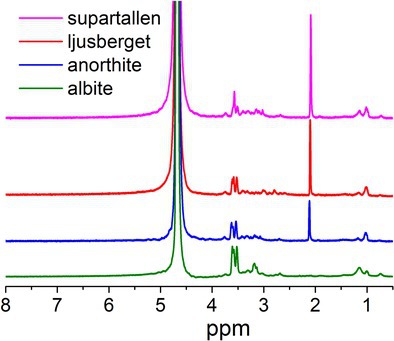

## 1 Introduction

A bitumen emulsion is complex in its molecular and phase compositions. Its main components are bitumen, water, and emulsifier. It is a continuum of hydrocarbons with low percentages of heteroatoms, including saturated and naphthenic hydrocarbons; polyaromatics; polar aromatics, including phenols and carboxylic acids; high‐molecular‐weight asphaltenes; and heterocyclic compounds [1, 2, 3, 4]. Most bitumen may also contain organosulfur compounds, nitrogen, oxygen, phosphorus, and traces of metals such as vanadium, nickel, and iron. There is a point of view that the fractions can be considered as “bitumen components,” while the fractions are generally very heterogeneous [1, 5]. The most common concept of bitumen is that it is a colloid comprising solid asphaltenes as the dispersed phase and liquid components (maltenes) as the dispersion medium [2, 6]. Bitumen solubility is a fundamental aspect of bitumen technology [1, 5, 6]. Bitumen is soluble in chloroform along with other solvents like carbon disulfide, carbon tetrachloride, toluene, and trichloroethylene [6], while its components demonstrate a different solubility in organic solvents. Maltenes are soluble in aliphatic solvents (*n*‐pentane, *n*‐heptane), while asphaltenes, which are more polar, polyaromatic hydrocarbons with high molecular weight, precipitate upon dilution with *n*‐alkanes but exhibit solubility in aromatic solvents like toluene. The poor solubility of asphaltenes in aliphatic solvents is used for asphaltene precipitation from crude oils. Hybrid solvents are also used to desorb bitumen components from mineral surfaces [7] and reveal desorption mechanisms.

By the phase composition, a bitumen emulsion is a dispersion of bitumen droplets (dispersed phase) in an aqueous continuous phase, stabilized by the addition of an emulsifier [4], while the particles may also contain trapped micro‐droplets of water [8, 9]. The emulsions are usually prepared as genuine emulsions at high temperatures but applied as solid dispersions at ambient temperatures [10]. In an industrial process (e.g., during road construction), the emulsion must break over the surface of a range of mineral aggregates in a controlled manner [10].

In road construction, bitumen products are typically applied in conjunction with mineral aggregates. The strong adhesion of bitumen to the mineral aggregates allows it to act as a binder [11, 12], while the aggregate provides mechanical strength. Most mineral aggregates are acidic. Acidic mineral aggregates such as silicates carry a net negative charge under normally encountered pH conditions [13]. An aggregate containing 66% or more of silica is classified as acidic; also, dry and wet silicate surfaces are negatively charged [14]. Aggregates containing calcareous mineral deposits, although positively charged when dry, become negatively charged when wet with water [15]. Acidic aggregates have a high affinity towards cationic bitumen emulsions and result in good adhesion when mixed together. The role of the surfactant in these systems is to make the polar mineral aggregate surface hydrophobic, thus making it easier for the non‐polar bitumen to adhere [16].

There are several stages of formation of a bitumen–mineral composition [10]. Initially, cationic bitumen emulsions break by means of physicochemical interactions between the emulsion droplets and the mineral aggregate. Cationic surfactant molecules in the continuous phase of the emulsion adsorb onto the solid aggregate surface. As a result, the modified surface of the aggregate is rendered ideal for wetting by the bitumen released in the breaking process. Local water absorption into the mineral aggregate and surfactant adsorption onto the aggregate lead to a decrease in the concentration of surfactant molecules available to stabilize the emulsion. Consequently, the surface of the mineral aggregate changes due to surfactant adsorption and bitumen deposition. Adsorption of the surfactant from the aqueous phase onto the mineral aggregate surface leads to a decrease in the concentration of surfactant in the bulk aqueous phase, which is likely to drop below its critical micelle concentration. To restore the equilibrium of the bulk aqueous phase, surfactant molecules from the oil–water interface migrate to the aqueous phase, reducing the number of surfactant molecules on the bitumen droplets. The bitumen droplets would then no longer be separated by electrostatic repulsive forces, resulting in coagulation and coalescence. Finally, the bitumen droplets adhere to the mineral surface already modified by the surfactant and then combine with each other forming the bitumen film [17]. Therefore, the bitumen emulsion–mineral interface serves as a marker for the processes occurring in the formation of a bitumen cold mix asphalt layer and it influences the kinetics and quality of the final product.

Nuclear magnetic resonance (NMR) is a convenient method to study processes at the molecular level in different heterogeneous systems, including oil [9, 18, 19] and bitumen [9, 20]. NMR spectra provide valuable information about the chemical composition, physical transformations, and chemical reactions in the system of study. It is challenging to apply NMR to study heterogeneous (porous or dispersed) systems. Firstly, it is known that in heterogeneous systems, such as liquid filling porous or dispersed media, placed in a homogeneous magnetic field, a “jump” in the magnetic susceptibility, taking place on the liquid–solid border, leads to the appearance of a background gradient [21]. This results in the broadening of NMR resonance lines, enhanced NMR relaxation, and NMR diffusion artifacts [21, 22]. The presence of background gradients generally obstructs the observation of the molecular events occurring in liquid layers and at the liquid–solid interface. Additionally, most minerals used in road construction contain ferromagnetic and strongly paramagnetic particles with compounds of iron, nickel, cobalt, copper, and rare earth elements. Even the addition of small amounts of these minerals to the bitumen dramatically enhances NMR relaxation of protons [9] and thus does not allow the use of NMR to study adhesion phenomena and kinetic processes in bitumen–mineral compositions.

In this study, we used a ^1^H NMR approach based on the extraction of a specified bitumen emulsion component that forms the interface layer together with the surfactant normally present in the emulsion. The interface layer composition may be controlled by the composition of the contacting mineral surface [23]. Our idea is to remove the “bulky” part of the emulsion (which does not interact with the surface) by flushing with an appropriate solvent and then extract and study the interfacial bitumen‐mineral component. It is known that dichloromethane and toluene dissolve most of the bitumen components. Therefore, these solvents may be used to dissolve all bitumen components of the bitumen–mineral–aggregate system, including those of the interface layer. *n*‐hexane, like other *n*‐alkanes, is a “weaker” solvent, which dissolves only the maltene fraction of the bulk bitumen. For this reason, we used *n*‐hexane in our study of the interfacial interactions between bitumen, surfactants, and Na/Ca plagioclase mineral surfaces. After flushing off the “bulky” bitumen, the interface layer was extracted and collected into an NMR glass tube. Experiments showed that the magnetic components of minerals are not extracted together with the interface layer components of the bitumen emulsion and, therefore, do not interfere with NMR experiments. This approach turns out to be informative with respect to some aspects of the mineral–bitumen emulsion interactions concerning the formation of the bitumen–mineral interface. The ^1^H NMR method also allows the analysis of compositions with a broad range of minerals, including ferromagnetic and paramagnetic ones. Thus, the applicability of NMR to the study of bitumen–mineral systems can be somewhat expanded.

## 2 Materials and Methods

### 2.1 Materials

A slow‐breaking bitumen emulsion of the EN‐grade: C67B4‐160/220 (according to EN 13808) was kindly provided by Nynas AB. The bitumen emulsion used is cationic. This type of emulsion breaks down on contact with the (mostly negatively charged) aggregates, resulting in significantly better adhesion. This type of emulsion in combination with a breaking additive is used in cold‐mix asphalt technology.

*n*‐Hexane (ReagentPlus, ≥ 99%) was purchased from Sigma‐Aldrich. Deuterated water (D_2_O, 99.90% D) was purchased from Eurisotop (St. Aubin Cedex).

Four minerals were used: albite (Brazil), anorthite (Kenya), and Supartallen and Ljusberget (kindly provided by NCC). Both albite and anorthite belong to the mineral series plagioclase, but with different contents of sodium and calcium in their compositions. The series of plagioclases ranges from albite to anorthite with the elemental composition of (Na, Ca) ranging from a pure sodium plagioclase, NaAlSi_3_O_8_, to a pure calcium plagioclase CaAl_2_Si_2_O_8_, where sodium and calcium atoms can substitute each other in the mineral's crystal lattice structure. Thus, albite contains mainly NaAlSi_3_O_8_ (90%–100% of Na), while anorthite contains mainly CaAl_2_Si_2_O_8_ (90%–100% of Ca). Minerals from the mines Ljusberget and Supartallen also contain impurities including paramagnetic Fe (major) and Ni, Mn, Co, and rare earth elements (REEs) (minor).

Supartallen and Ljusberget minerals are widely used in the asphalt industry in Northern Sweden. Their mineral compositions are shown in Table 1.

**TABLE 1:** Mineral compositions of minerals from mines Supartallen and Ljusberget (volume %) [24].

	Supartallen	Ljusberget
Quartz	31.5	87.7
Potash feldspar/alkali feldspar	13.9	6.2
Plagioclase	44.0	0.7
Biotite	9.0	0.0
Diverse (not identified)	1.6	5.4

The content of ferromagnetic elements in a mineral is especially important when they are being studied by NMR. Therefore, the element contents of the mineral samples were analyzed by semi‐quantitative screening using ICP‐MS performed by “ALS Scandinavia AB.” The obtained data indicate the contents of iron, cobalt, and nickel, as shown in Table 2.

**TABLE 2:** Contents of some elements in mineral samples studied according to semi‐quantitative screening analysis by ICP‐MS.

	Fe, mg/kg	Co, mg/kg	Ni, mg/kg	Na, mg/kg	Ca, mg/kg	Ca/Na
**1**	**2**	**3**	**4**	**5**	**6**	**7**
Albite	220	0.058	5.0	89⋅10^3^	1.8⋅10^3^	0.02
Anorthite	3.3⋅10^3^	0.15	0.64	56⋅10^3^	13⋅10^3^	0.23
Supartallen	22.3⋅10^3^	5.7	4.9	32⋅10^3^	18⋅10^3^	0.56
Ljusberget	3.2⋅10^3^	1.1	20	1.4⋅10^3^	4⋅10^3^	2.85

Mineral aggregates were milled and fractionated with brass sieves. Fractions of the size 1 mm < *d* < 1.5 mm were collected and used in experiments. The porosity of albite, anorthite, Supartallen, and Ljusberget was analyzed by the Brunauer–Emmett–Teller (BET) technique. N_2_ absorption was analyzed at liquid nitrogen temperature. For all four samples, no pores in the sensitive to BET range of 0.3–200 nm were found.

In some experiments, we used a VWR ultrasonic cleaner water bath at 45 kHz, 80 W, to treat the samples.

### 2.2 NMR Technique

NMR spectra measurements were executed on a Bruker Avance III/Aeon 400WB (Bruker BioSpin AG) NMR spectrometer with a working frequency for protons of 400.21 MHz (induction of the static magnetic field 9.4 T). ^1^H NMR spectra were obtained by a Fast Fourier Transformation (FFT)of the free induction decay (FID) following the 90° radiofrequency pulse (90°‐*acq*). A sample was placed in a standard 5‐mm glass sample tube. The tube was closed with a plastic stopper to avoid air contact. Bulk bitumen emulsion and bitumen solution in deuterated chloroform (CDCl_3_) were used as references in the ^1^H NMR experiment.

Pulsed gradient spin echo‐nuclear magnetic resonance (PGSE‐NMR) measurements were performed with a PGSE‐NMR probe Diff50 (Bruker). Prior to the measurements, the sample was equilibrated at a specific temperature for 15 min. The diffusional decays (DD) were recorded using the stimulated echo (StE) pulse train. For single‐component diffusion, the form of the DD can be described as [25]:

(1) $$A\left(\tau ,\tau_{1},g,\delta \right)\propto \exp\left(-\frac{2\tau }{T_{2}}-\frac{\tau_{1}}{T_{1}}\right)\exp\left(-\gamma^{2}\delta^{2}g^{2}Dt_{d}\right).$$

Here, *A* is the integral intensity of the NMR signal, *τ* is the time interval between first and second radiofrequency pulses, *τ*_1_ is the time interval between second and third radiofrequency pulses. *γ* is the gyromagnetic ratio for protons; *g* and *δ* are the amplitude and the duration of the gradient pulse, respectively; *t*_d_ = (*Δ* − *δ*/3) is the diffusion time; *Δ* is the time interval between two identical gradient pulses; and *D* is the diffusion coefficient. In the measurements, the duration of the 90° pulse was 7 μs, *δ* was in the range of 1–3 ms, *τ* was in the range of 3–5 ms, and *g* varied from 0.06 up to the maximum of the gradient amplitude, 29.73 T·m^−1^. Diffusion time was 20 ms. The repetition time during the accumulation of signal transients was 5 s.

### 2.3 Preparation of Samples for NMR Spectroscopy

**FIGURE 1:**
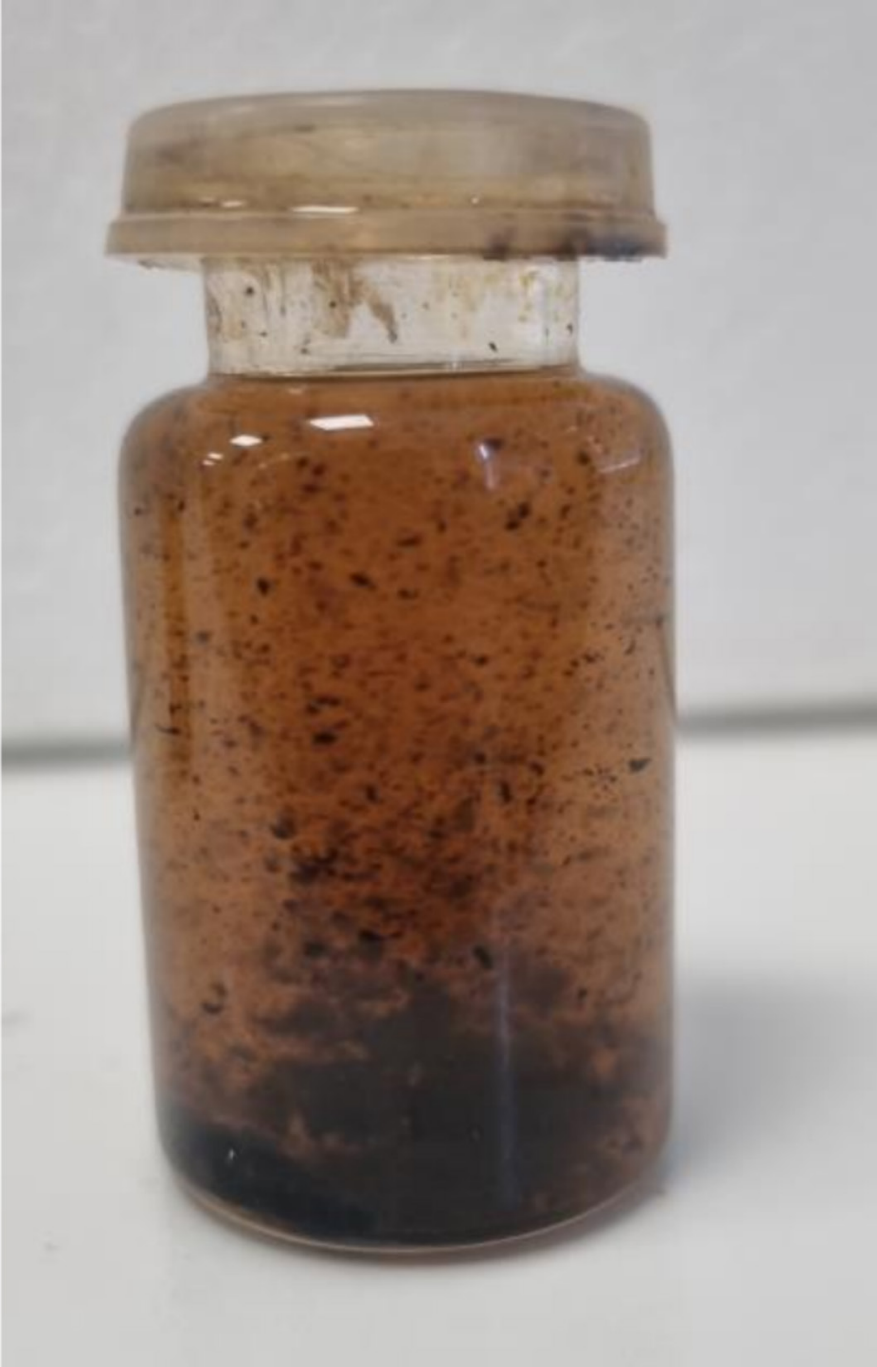
Albite–bitumen emulsion composition after water removal and during *n*‐hexane washings. The maltene fraction of the bitumen can be dissolved, while the asphaltene fraction forms flakes. Both fractions are removed from the system during the preparation stages.

**FIGURE 2:**
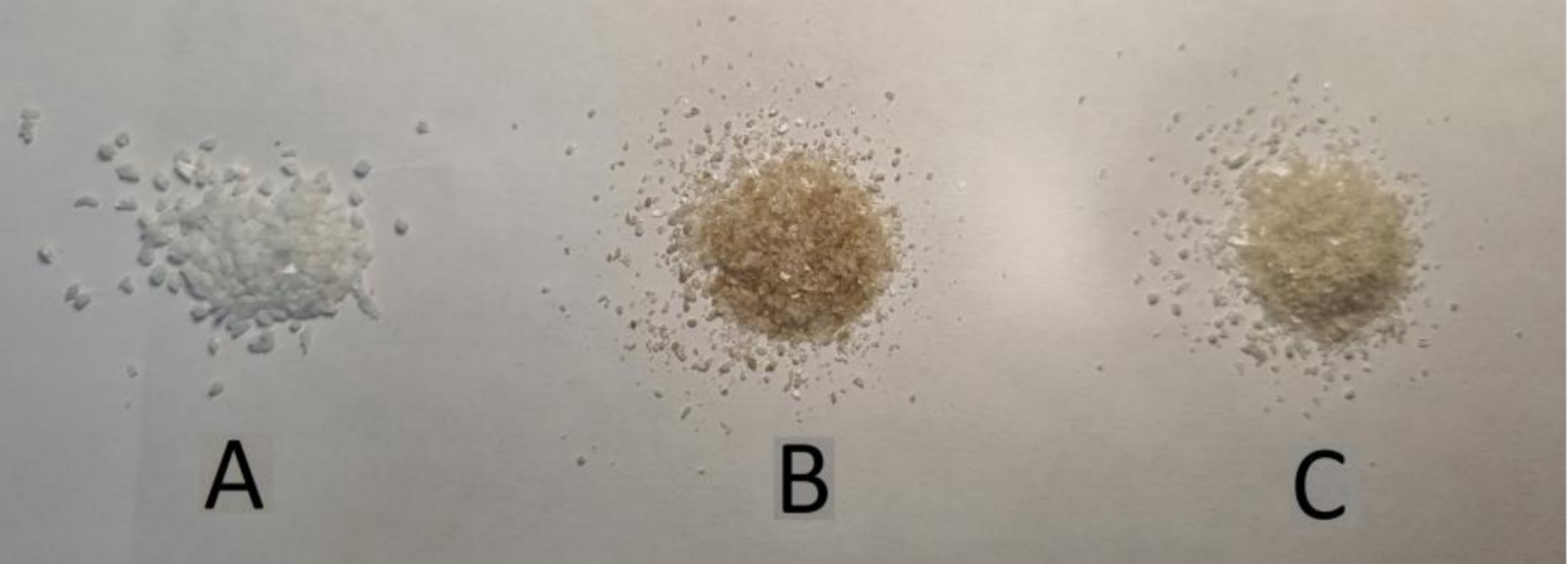
Albite particles before mixing with the bitumen emulsion (A), after the final step of washing with *n*‐hexane (B) and water (C).

To prepare bitumen–mineral compositions ~0.5 g of a mineral was mixed with 300 μL of the bitumen emulsion, closed and conditioned at 60°C for 1 h. Normally, the bitumen emulsion is stable at 60°C–80°C. The sample was then cooled to 22°C. Excess condensed water on the walls of the vial was subsequently removed by wiping, drying in a flow of N_2_, and subjecting the sample to a vacuum. Bitumen was collected by numerous washings using *n*‐hexane until the washing solvent became colorless. It is known [1] that *n*‐hexane dissolves the maltene fraction, while the asphaltene fraction is not soluble in it. Upon addition of the solvent, we observed coloration (from brown to black color) of the liquid as well as the formation of black flakes of a solid (Figure 1). The latter can be related to asphaltene colloidal particles not soluble in *n*‐hexane. All the “after‐washing” liquid containing flakes was disposed off (not analyzed by NMR). After washing, residual traces of the solvents were removed by drying the samples of mineral particles in a vacuum (50 Pa) for 12 h. Typical crystals (grains) of a mineral (albite as an example) before the treatment and after consecutive washings in *n*‐hexane are shown in Figure 2A,B. Then, 500 μL of D_2_O was used to wash out (during ~2 min) the surface layers of dry mineral grains. In some cases, this was done by a gentle shaking of the sample tube. In some cases, ultrasound water‐bath sonication was also applied to the latter sample. After washing the samples with D_2_O, the solutions were collected in NMR tubes and analyzed by ^1^H NMR spectroscopy. The mineral (albite) grains lightened after the treatment with water (Figure 2C).

## 3 Results and Discussion

**FIGURE 3:**
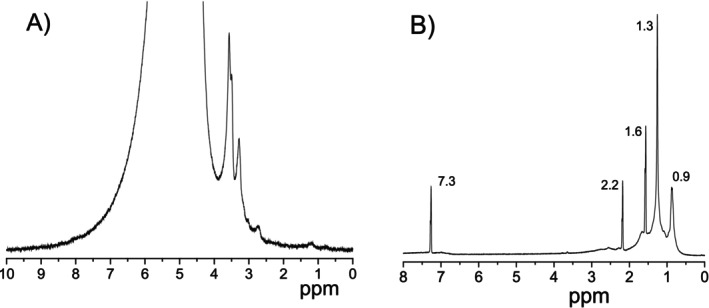
^1^H NMR spectra of (A) the original bitumen emulsion. The resonance line of the water proton signal at ~4.6 ppm is cut off to provide better visibility of the NMR from other (organic) components. (B) Solution of the bitumen acquired from the emulsion and then dissolved in CDCl_3_. A line at 7.3 ppm corresponds to residual CDCl_3_ protons.

Figure 3A shows the ^1^H NMR spectrum of the bulk emulsion, which is similar to the previously reported one by Filippov et al. [9]. Different expansions of the spectrum are shown in Figure S1 of the Supporting Information (SI). Except for the strong water proton signal at ~4.6 ppm (resonance line not shown), there are also several signals, which can be related to the bitumen and surfactant components. The latter signals appear only partially in the spectrum, because of their short *T*_2_ relaxation times in the condensed state with a high viscosity (bitumen droplets) and a low rotational mobility of the droplets. Most intense resonance lines in the range from 2.5 to 4 ppm can be putatively assigned to water‐soluble (partially water‐soluble) components, such as surfactants present in the bitumen emulsion.

Figure 3B shows the ^1^H NMR spectrum of the solution of the bitumen fraction of the original bitumen emulsion obtained after removing water from the emulsion and then dissolved in deuterated chloroform. Different expansions of the spectrum are shown in Figure S2 of the SI. In the range 0–4 ppm, the spectrum demonstrates signals characteristic of bitumen components, such as asphaltenes and maltenes [26, 27, 28, 29, 30, 31, 32, 33, 34], while water‐soluble components observed in Figure 3A are not visible in Figure 3B. Signals at 0.9, 1.3, and 1.6 ppm were assigned to aliphatic protons in –CH_3_ and CH_2_ groups [27, 28, 32]. The signal near 2.2 ppm was assigned to aliphatic hydroxyls [33] and also to ‐CH_3_ attached to aryl rings [26, 27, 28]. The signal at 7.3 ppm is assigned to residual protons of the solvent, deuterated chloroform. Therefore, Figure 3B shows complexity of the ^1^H NMR spectrum corresponding to bitumen, which is not seen in Figure 3A.

**FIGURE 4:**
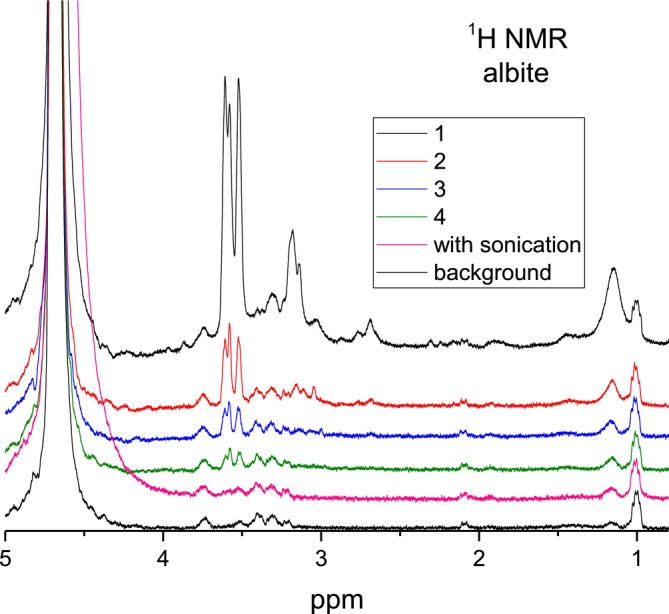
^1^H NMR spectrum in the range of 5–0.7 ppm after several consecutive flushes with D_2_O of an albite–bitumen emulsion initially washed with *n*‐hexane. Each flushing contains 500 μL of D_2_O.

Let us discuss ^1^H NMR spectra obtained from flushes of bitumen emulsion–mineral compositions starting with the albite mineral. The sample of the bitumen–albite composition was prepared, and then water and “bulky” bitumen were consequently removed with *n*‐hexane as described in Section 2 (see Figure 2). After that, we flushed the sample of albite particles (Figure 2B) with D_2_O. We performed five consecutive D_2_O flushes with gentle mixing and the sixth flush assisted with sonication in the ultrasonic cleaning water bath (5 min at 293 K). Consequently, this is D_2_O with water‐soluble components of the bitumen emulsion remaining after extraction of the part of the emulsion with *n*‐hexane. Spectra of the solutions are shown in Figure 4. ^1^H NMR spectra of flushes from neat albite do not show any NMR signals in the range of interest, 0–3.7 ppm, apart from the broad background (spectra not shown).

Figure 4 shows that the ^1^H NMR spectra do not contain signals in the same spectral range as in the bulk bitumen emulsion (compare with Figure 3A). Water‐soluble components give resonance lines at ~1.2, 2.7, 3.2, and 3.5–3.7 ppm. The consecutive flushing of the mineral leads to a continuous decrease in the intensity of the NMR spectral lines. Therefore, D_2_O flushing subsequently removes the water‐soluble component from the surface of the albite mineral. No resonance lines characteristic to the bitumen were observed in these spectra (compare with the spectrum in Figure 3B).

**FIGURE 5:**
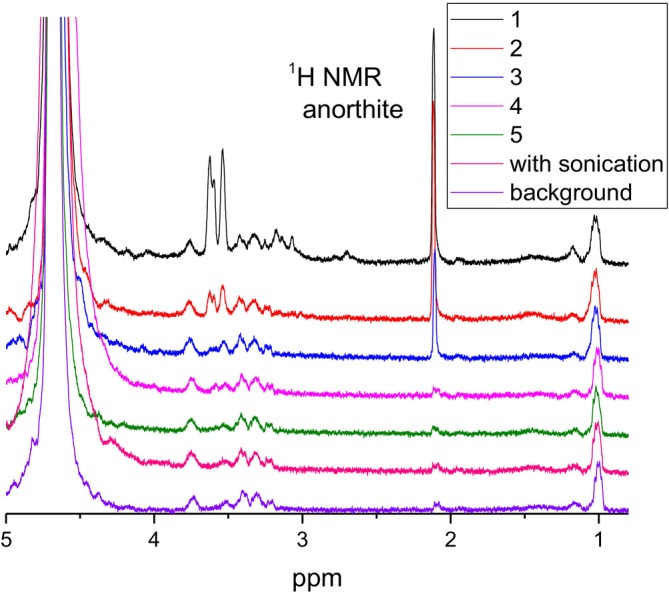
^1^H NMR spectra of several consecutive flushes with D_2_O of an anorthite–bitumen emulsion sample initially washed with *n*‐hexane. Each flushing contains 500 μL of D_2_O.

**FIGURE 6:**
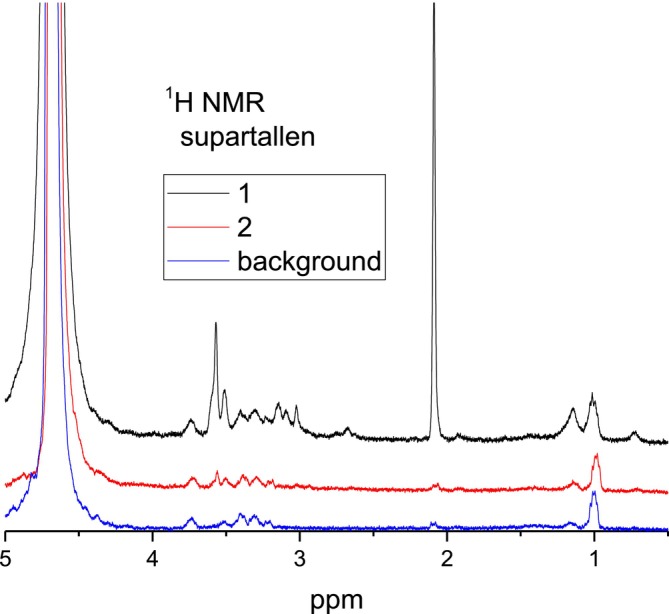
^1^H NMR spectra of two consecutive flushes with D_2_O of a Supartallen–bitumen emulsion sample initially washed with *n*‐hexane. Each flushing contains 500 μL of D_2_O.

**FIGURE 7:**
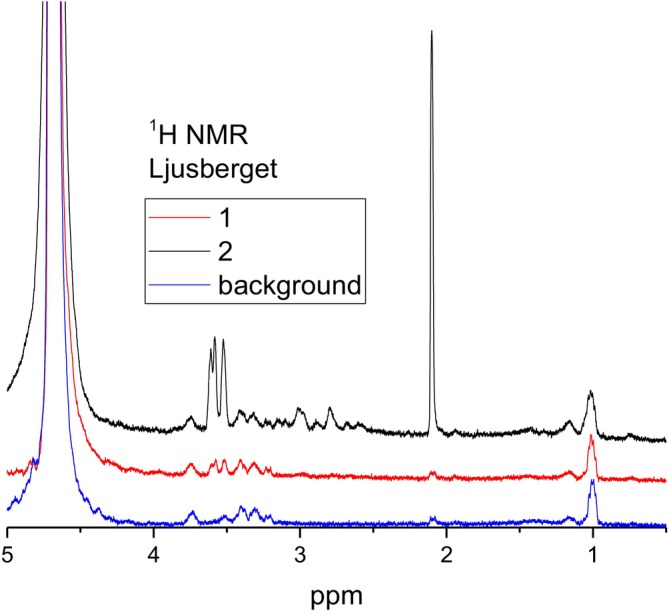
^1^H NMR spectra of two consecutive flushes with D_2_O of a Ljusberget–bitumen emulsion sample initially washed with *n*‐hexane. Each flushing contains 500 μL of D_2_O.

^1^H NMR spectra for the other three minerals are shown in Figures 5, 6, 7. There are similarities as well as differences in the NMR spectra obtained for the samples prepared with these minerals. Firstly, independent of whether the used mineral contains (anorthite, Supartallen, Ljusberget) or does not contain (albite) magnetic impurities, the ^1^H NMR spectra demonstrate rather sharp resonance lines. This means that neither magnetic particles, nor leached paramagnetic ions from minerals are present in the analyzed D_2_O solution collected and analyzed after flushing of the mineral particles. This is not unexpected since the *n*‐alkane used is not polar. However, it is critical for NMR studies. Secondly, the consecutive flushing removes the water‐soluble component from the surfaces of minerals in all cases. The final ^1^H NMR spectra after multiple rounds of flushing are similar to each other and are the same as the ^1^H NMR spectrum of the background (compare spectra “with sonication” in Figures 4 and 5 with “2” in Figures 6 and 7). The experiments were repeated several times. The results were reproducible.

**FIGURE 8:**
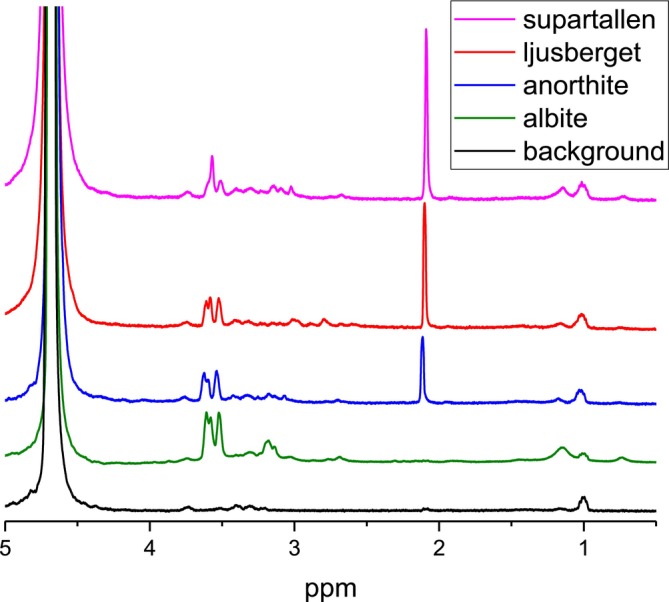
^1^H NMR spectra of the first flushing with D_2_O of the mineral–bitumen emulsion samples initially washed with *n*‐hexane.

However, the spectra of the first flushing are different, and they do change differently between the subsequent rounds of flushing for different samples of bitumen‐mineral compositions. This, evidently, shows the differences in the composition of the interface layer formed on the surfaces of the minerals and the different degrees of interaction of the bitumen emulsion with the mineral surfaces. ^1^H NMR spectra obtained after the first flushing with D_2_O are shown in Figure 8 for comparison.

As can be seen in the ^1^H NMR spectra presented in Figure 8, the water‐soluble bitumen emulsion fraction obtained from the interface layers of minerals has organic species with chemical groups with resonances at 1.2 ppm (for albite and anorthite), 3.2 ppm (for albite), and 3.5–3.7 ppm (for all four minerals). Integral intensities of resonance signals at 1.2 and 3.5–3.7 ppm do change in concert. Therefore, they can putatively be assigned to one and the same specific organic molecule. The same NMR signals appeared in the ^1^H NMR spectrum of the original bitumen emulsion (Figure 3A), but not in bitumen (Figure 3B) and, thus, can be related to surfactants stabilizing the bitumen emulsion. From the chemical shift values, we can assign signals at ~1.2 ppm to –CH_3_ chemical groups and the signals at 3.6–3.7 ppm to –CH_2_–OH and –CH_2_–CH_2_–OH chemical groups [22]. The ^1^H NMR signal at ~2.1 ppm is characteristic for asphaltenes, while it is not observed in maltenes [31, 32]. The resonance line at 2.1 ppm in asphaltenes was previously assigned to protons of aliphatic hydroxyls in the α‐position next to the aromatic ring [33] and to the –CH_3_ groups directly bonded to aryl rings [26, 27, 28], while it is not observed in maltenes [31, 32]. Asphaltenes with the latter (highly mobile) aryl‐CH_3_ groups are dominating in D_2_O flushes, since no other aliphatic protons (in the spectral range from 0 to 1.5 ppm corresponding to CH_2_ and CH_3_ groups in alkyl chains) are readily detected in ^1^H NMR spectra of the mineral–bitumen emulsion samples. Therefore, ^1^H NMR spectra shown in Figures 5, 6, 7, 8 revealed that a specific fraction of asphaltenes is adsorbed on surfaces of anorthite, Supartallen, and Ljusberget together with molecules of surfactants, while no asphaltenes, but only surfactants, are adsorbed on the albite surfaces.

It should be noted that the ^1^H NMR signal of asphaltenes at ~2.1 ppm is not present in the original bitumen emulsion (Figure 3A), but it appears in D_2_O flushes. Apparently, the water‐insoluble asphaltenes, being adsorbed on the mineral surfaces together with surfactants, may form water‐soluble associates.

Note that consecutive flushings with D_2_O does remove asphaltenes from mineral surfaces more readily for Supartallen (Figure 6) and Ljusberget (Figure 7) in comparison to anorthite (Figure 5), while surfactants are more easily removed from all three of these minerals. In addition, asphaltenes do not absorb on albite (Figure 4). The adsorption propensity of asphaltenes on mineral surfaces does correlate with the amount of calcium and Ca/Na ratio in these minerals (Table 2): The larger the amounts of calcium that are present in a mineral, the higher is the relative integral intensity of the resonance line at 2.1 ppm detected in the ^1^H NMR spectrum (see Figure 8). We did not analyze the influence of other elements of the studied plagioclase mineral series, such as aluminum, which can also influence bitumen absorption.

The size of a particle diffusing in a liquid is related to its diffusion coefficient *D* by the Stokes–Einstein equation for the motion of a solid sphere in a viscous medium:

(2) $$D=\frac{\mathrm{kT}}{f}=\frac{\mathrm{kT}}{6\mathrm{\eta \pi R}},$$

where *k* is the Boltzmann constant, *Т* is the temperature, *f* is the friction coefficient, *η* is the viscosity of the medium, and *R* is the hydrodynamic radius of the sphere. We measured the diffusivity of bitumen–surfactant associates obtained by washing anorthite, which are characterized by the ^1^H NMR spectrum in Figure 5 (curve 1). The measurement was performed at 293 K. Diffusion decay analysis was performed in the 4–0 ppm range and showed a non‐exponential form of the diffusion decay (see Figure S3 of the SI), which can be presented as a sum of two decays with slopes corresponding to diffusion coefficients ~2.4⋅10^−10^ and ~5.5⋅10^−11^ m^2^/s and apparent fractions 0.45 and 0.55, respectively. Values of the diffusion coefficients are quite close to those obtained earlier by Werkovits et al. [20] for bitumen fractions. We can estimate hydrodynamic sizes (diameters, 2⋅*R*) of the diffusing asphaltene–surfactant particles using Equation (2) and taking the viscosity of D_2_O as being equal to 1.25 cP at 293 K [35]. This gives values of 1.4 and 6.2 nm for the diameter (2⋅*R*) of moving asphaltene–surfactant particles, which is of the order of sizes of earlier obtained asphaltene aggregates [36] and bitumen fractions [20].

The interaction of bitumen with minerals plays an important role in the strength and durability of the road pavement. In the cold‐asphalt technology, the type of mineral used as a ballast material in asphalt appeared to influence the formation of the mineral–bitumen interface. This study revealed that the composition of the interface layer formed in the course of setting the bitumen emulsion depends on the elemental composition and specifically on the Ca/Na composition of ballast minerals: Calcium promotes stronger adsorption of asphaltenes on mineral surfaces that, in turn, has a decisive role for mechanical properties, consistency, and brittleness of asphalt. It is known that calcium hydroxide belongs to the class of anti‐strip agents [24]. It serves to consolidate fines and clays on the aggregate surface and to provide calcium binding sites for bitumen on the aggregate surface [37]. There are earlier experimental observations that a higher percentage of calcium promotes higher dispersive adhesion to bitumen [24, 36, 38]. A possible mechanism has been proposed by Ziyani et al. [38]. They analyzed the reactivity of three different aggregates (gneiss, diorite, and limestone) containing different percentages of Ca with a cold‐asphalt‐mixture cationic bitumen emulsion using a pH rise test. This analysis revealed that calcium was the main element released. The release of Ca in the solution has a consequence of the emulsion breaking and bitumen absorption. These findings can be taken into account in the road pavement design by selecting appropriate ballast minerals.

## 4 Conclusions

We propose a ^1^H NMR approach to study some aspects of specified bitumen emulsion–mineral interactions, which can be used even for minerals containing magnetic constituents. The approach is based on a delicate extraction of the bitumen emulsion components forming bitumen–mineral interface layers. First, both water and “bulky” bitumen were removed by washing the bitumen–mineral compositions with *n*‐hexane. Then the samples of mineral particles were repeatedly flushed with D_2_O and aliquots analyzed by ^1^H NMR. It was found that magnetic components of minerals or leached paramagnetic metal complexes and ions are not extracted from the interface layers of such treated minerals. This is expected, but it is critical for NMR applications in any system. Therefore, we pay special attention to this issue. The ^1^H NMR study was carried out on a range of samples prepared with the same bitumen emulsion and four different minerals (plagioclase minerals: albite and anorthite and two mineral compositions used in road construction in Northern Sweden; the latter two are from the mines Supartallen and Ljusberget). The study revealed that the interface layers contain different amounts of surfactants and asphaltenes depending on the mineral type. The adsorption propensity of asphaltenes on selected mineral surfaces does correlate with the amount of calcium in the mineral: more asphaltenes were detected in the interface layers on surfaces of studied minerals with a higher content of calcium. We observed trends demonstrating the capabilities of NMR spectroscopy in combination with special preparation of minerals containing magnetic impurities. More experiments with more specific sets of minerals are needed for more definitive conclusions.

Generally, our study shows the potential to use this method to apply NMR spectroscopy to the study of bitumen emulsion–mineral interactions, despite a high content of iron or other paramagnetic elements in minerals, which can obscure NMR measurements. A more detailed study and further development of the method, as well as its combination with other physicochemical methods, are currently underway in our laboratory and will be published elsewhere.

## Supporting information


**Figure S1:** mrc70056‐sup‐0001‐Supplementary_Material.docx. ^1^H NMR spectrum of the bitumen emulsion: (A) whole range and (B) magnified to better visualize resonance lines assigned to organic compounds.
**Figure S2:** mrc70056‐sup‐0001‐Supplementary_Material.docx. ^1^H NMR spectrum of flushing of the bitumen by CDCl_3_: (A) whole range and (B) magnified to better visualize resonance lines assigned to organic compounds. A signal at 7.3 ppm corresponds to residual protons in CD(H)Cl_3_.
**Figure S3:** mrc70056‐sup‐0001‐Supplementary_Material.docx. ^1^H NMR diffusion decay of the first D_2_O flushing of the sample of anorthite (Kenya)–bitumen emulsion. The sample was initially washed with *n*‐hexane to remove the bulk bitumen.

## Data Availability

The data that support the findings of this study are available from the corresponding author upon reasonable request.
