# Supplementary material for: Bitumen Emulsion—Mineral Surface Interactions: An NMR Study on the Interface Layer Composition
Source: Magn Reson Chem. 2025 Nov 14;64(2):170–8. doi: 10.1002/mrc.70056 (PMC12783950; doi:10.1002/mrc.70056)
Supplement: Supplementary file 1 — Figure S1: mrc70056‐sup‐0001‐Supplementary_Material.docx. 1H NMR spectrum of the bitumen emulsion: (A) whole range and (B) magnified to better visualize resonance lines assigned to organic compounds. Figure S2: mrc70056‐sup‐0001‐Supplementary_Material.docx. 1H NMR spectrum of flushing of the bitumen by CDCl3: (A) whole range and (B) magnified to better visualize resonance lines assigned to organic compounds. A signal at 7.3 ppm corresponds to residual protons in CD(H)Cl3. Figure S3: mrc70056‐sup‐0001‐Supplementary_Material.docx. 1H NMR diffusion decay of the first D2O flushing of the sample of anorthite (Kenya)–bitumen emulsion. The sample was initially washed with n‐hexane to remove the bulk bitumen. [file MRC-64-170-s001.docx]

Supporting Information

**Bitumen emulsion – mineral surface interactions: An NMR study on the interface layer composition**

Andrei Filippov,^a^* Hilde Soenen,^b^ Oleg N. Antzutkin^a^

^a^Chemistry of Interfaces, Department of Civil and Environmental Engineering, Luleå University of Technology, Luleå SE-97187, Sweden

^b^Nynas N.V., 171 Groenenborgerlaan, Antwerp 2020, Belgium

|  |  |
| --- | --- |

**Fig. S1.** ^1^H NMR spectrum of the bitumen emulsion: A) whole range and B) magnified to better visualise resonance lines assigned to organic compounds.

|  |  |
| --- | --- |

**Fig. S2.** ^1^H NMR spectrum of flushing of the bitumen by CDCl_3_: A) whole range and B) magnified to better visualise resonance lines assigned to organic compounds. A signal at 7.3 ppm corresponds to residual protons in CD(H)Cl_3_.

**Fig. S3.** ^1^H NMR diffusion decay of the first D_2_O flushing of the sample of anorthite (Kenya)-bitumen emulsion. The sample was initially washed with *n*-hexane to remove the bulk bitumen.
